# Thyroid functions of neonates with Down syndrome

**DOI:** 10.1186/1824-7288-38-44

**Published:** 2012-09-17

**Authors:** Dilek Sarici, Mustafa Ali Akin, Selim Kurtoglu, Tamer Gunes, Mehmet Adnan Ozturk, Mustafa Akcakus

**Affiliations:** 1 Department of Pediatrics, Division of Neonatology, Faculty of Medicine, Erciyes University, Kayseri, 38039, Turkey; 2 Department of Pediatrics, Division of Neonatology, Faculty of Medicine, Akdeniz University, Antalya, 07070, Turkey

**Keywords:** Down syndrome, Hyperthyrotropinemia, Newborn

## Abstract

**Background:**

We aimed to evaluate thyroid functions and volumes and detect abnormalities in 80 neonates with Down syndrome.

**Methods:**

Data about free triiodothyronine, free thyroxine, thyroid stimulating hormone, thyroglobulin and urinary iodine levels, and ultrasonographic thyroid volume were collected.

**Results:**

Abnormal thyroid function tests were detected in 53.8% of the patients (n = 50) and these were hyperthyrotropinemia, hypothyroidism, iodine deficiency and iodine overload in 32, 2, 12 and 4 patients, respectively. Thyroid volumes were assessed in 36 patients and a total of 17 abnormalities were detected (7 hypoplasia, 3 agenesis and 7 goiter). In patients with hyperthyrotropinemia mean thyroid volume was significantly greater and mean TSH was significantly higher when compared to the patients without hyperthyrotropinemia.

**Conclusion:**

Neonatal screening by thyroid function tests in Down syndrome should be performed to prevent further intellectual deterioration and improve overall development. In the neonatal period, the risk of hyperthyrotropinemia should be kept in mind.

## Introduction

Among the endocrinopathies associated with Down syndrome (DS), thyroid gland disorder is the best-known endocrine abnormality. Thyroid disorders include subclinical and overt hypothyroidism, hyperthyroidism, and positive thyroid antibodies. Subclinical hypothyroidism(SH) is usually characterized by hyperthyrotropinemia and marked by elevated TSH and normal T4 levels. Approximately half of all children with DS may have an elevated TSH level with normal T3 and T4 levels, which suggest subclinical hypothyroidism [1].

The clinical manifestations of hypothyroidism are so nonspecific that it may be attributed to the DS itself. Thyroid hormones are necessary with respect to brain development, and therefore, thyroid disorders should be detected immediately. In this study we thus aimed to evaluate thyroid functions and detect abnormalities of thyroid hormone profile in neonates with DS.

## Methods

This retrospective study comprised 80 neonates with DS who were admitted to the Department of Neonatology of Erciyes University. All patients had clinical characteristics of DS and the diagnosis was confirmed in 41 of them by cytogenetic studies. The median admission age of the patients was 9.6 ± 7.2 days (1–28 days).

Study data were collected from the patients’ medical records. Demographic data about the mother and infant characteristics were recorded. Thyroid function tests, including free triiodothyronine (FT3), free thyroxine (FT4), thyroid stimulating hormone (TSH), thyroglobulin (TG) and urinary iodine level were measured when the baby was first admitted to our unit. FT3 and FT4 were measured by radioimmunoassay (The Radiochemical Centre, Amersham, U.K.), and TSH (The Radiochemical Centre, Amersham, U.K.) and TG (CIS Bio international, Cedex, France) were determined by using a sensitive immunoradiometric assay. Urinary iodine level was measured by Sandell-Kolthoff method. Urinary iodine levels were accepted as deficient, normal, and overloaded according to the following levels: <10 μg/dl, 10–20 μg/dl, >20 μg/dl, respectively. TSH was considered elevated if its level was higher than 20 and 10 mU/L in patients aged from newly born to one week and from eight days to one month, respectively [2]. TSH was considered suppressed if its level was lower than 0.4 mU/L. Primary hypothyroidism was recognized with low FT4 and elevated TSH levels, whereas SH was diagnosed by elevated TSH and normal FT4 levels. Hyperthyroidism was diagnosed by high FT4 and suppressed TSH levels. On the basis of these laboratory findings patients were grouped as euthyroid, hyperthyroid, hypothyroid or subclinical hypothyroid. Thyroid ultrasonography (USG) was performed by an experienced pediatric radiologist, and thyroid volumes of the patients were evaluated according to the study of Kurtoglu et al. [3]. The study was approved by the institutional ethics committee. Written informed consent was obtained from the parents of the patients for publication of this report.

Descriptive central statistics were given as means and medians, and dispersion was given as SDs and ranges. Because of the non-normal distribution of data, Wilcoxon non-parametric statistics were used for analyses. Four-field tables were tested using a Fisher’s exact test. A p-value of less than 0.05 was considered statistically significant. Statistical analyses were done using Student’s t-test and Mann–Whitney test. Spearman’s rank correlation was used in correlation analyses.

## Results

Hyperthyrotropinemia, iodine deficiency, iodine overload and hypothyroidism were detected in 32, 12, 4 and 2 of the patients, respectively. Results of the thyroid functions are given in Table 1. Unfortunately, not all the patients had urinary iodine levels measured and thyroid volumes recorded. Urinary iodine level was measured in 28 out of the 80 patients (Table 1), whereas thyroid volumes were measured in 36 of the 80 patients (Table 2). Hyperthyrotropinemia was associated with iodine deficiency in 5 (17.8%) and iodine overload in 2 (7.1%) of 28 patients**.** The median thyroid volume and TSH level of patients with hyperthyrotropinemia were statistically significantly higher in comparison to those without hyperthyrotropinemia (p = 0.01 and p = 0.001, respectively). There were no significant differences regarding the mean FT4, FT3 and TG levels between the patients with and without hyperthyrotropinemia (p = 0.08, p = 0.84 and p = 0.055, respectively) (Table 1).

**Table 1:** Patients’ thyroid functions, urine iodine levels and thyroid volumes

	**All cases (n = 80)**			**Normal thyroid function (n = 37)**			**Hyperthyrotropinemia (n = 32)**			p value*
	**n**	**Mean ± SD**	**Median (min-max)**	**n**	**Mean ± SD**	**Median (min-max)**	**n**	**Mean ± SD**	**Median (min-max)**	
**TSH (mU/L)**	80	24.8 ± 30.9	12.4 (0.08 ± 127.7)	37	7.1 ± 4.8	5.9 (0.08-16.2)	32	45.6 ± 33.4	34.7 (10.1-127.7)	0.001
**FT4 (pg/ml)**	80	13.6 ± 4.3	13.5 (5–26.4)	37	14.4 ± 3.7	14.1 (7.8-25.1)	32	12.5 ± 3.4	13.3 (7.2-22.5)	0.08
**FT3 (pg/ml)**	79	2.6 ± 0.9	2.5 (0.01-5.45)	36	2.6 ± 0.6	2.7 (1.1-3.8)	32	2.6 ± 1.1	2.5 (0.01-5.4)	0.84
**Thyroglobulin (ng/ml)**	62	102.3 ± 104.3	59.5 (1.9-510.8)	25	67.2 ± 51.9	49.4 (6.5-199)	27	142.4 ± 135.5	89.2 (4.4-510.8)	0.055
**Urine iodine (μg/dl)**	28	12.6 ± 12.3	10 (1–59)	3	13.6 ± 1.5	14 (12–15)	15	11.5 ± 10.3	10.2 (1–37)	-
**Thyroid volume (ml)**	36	0.55 ± 0.27	0.51 (0.20-1.51)	8	0.43 ± 0.14	0.42 (0.24-0.63)	22	0.64 ± 0.29	0.56 (0.31-1.51)	0.01

*: p values reflect the comparison values between the cases with and without hyperthyrotropinemia (normal thyroid function).

**Table 2:** Results of thyroid ultrasonography in the patients

**Thyroid volume**	**All cases**		**Normal thyroid function**		**Hyperthyrotropinemia**	
	**n = 36**	**%**	**n = 8**	**%**	**n = 22**	**%**
**Normal**	19	53.4	4	50	13	59
**Hypoplasia**	7	19.6	2	25	4	18.1
**Agenesis**	3	8.4	1	12.5	0	0
**Goiter**	7	19.6	1	12.5	5	22.7

Thyroid volumes showed hypoplasia in 4 (18.1%) and goiter in 5 (22.7%) of 22 patients with the diagnosis of hyperthyrotropinemia (Table 2). Thyroid agenesis was detected in 3 patients; all of them had normal thyroid functions, however two of them had iodine deficiency.

## Discussion

In a longitudinal study of 85 patients with DS (aged between 0 to 25 years) and without congenital hypothyroidism who were followed up for ≤15 years, 30 developed thyroid dysfunction, and 28 of them had hypothyroidism and the other two had hyperthyroidism [4]. Some children with DS demonstrate a persistently elevated TSH level for at least several years without developing overt hypothyroidism, whereas others revert to normal.

There are very few studies about the thyroid functions of newborns with DS. Van Troutsenburg et al. evaluated the course of thyroid function tests in infants with DS during the first 24 months and determined that concentrations of TSH, FT4, thyroglobulin were high, low and normal in the reference interval, respectively [5]. They claimed a direct relationship with the trisomic state of chromosome 21, hypothetically through genomic dosage imbalance of dosage-sensitive genes interfering with thyroid hormone production [5]. Myrelid et al. showed that 73 infants with DS had a high mean TSH level (7.0 ± 7.45 mU/L), especially in males, during neonatal screening, and 22 thyroxine-treated children were found in hospital records; 7 (3 female) with hypothyroidism, 6 children (3 female) with subclinical hypothyroidism, 9 children (5 female) with normal TSH levels and T4 levels close to the lower reference limit, however clinical signs could be related to hypothyroidism [6]. Karlsson et al. did not find any gender difference in thyroid dysfunctions [4] as also we observed in our study.

The present study demonstrated hyperthyrotropinemia in 32 of the 80 newborns with DS. The mean TSH level (45.6 ± 33.4 mU/L) was statistically significantly higher in cases with hyperthyrotropinemia in our study, and this level was also higher than that of the study of Myrelid et al. [6]. This difference may have resulted from our patients’ different gestational ages and the difference in the evaluation time at different postnatal ages.

A recent Turkish study showed that 28% of individuals with DS had abnormal thyroid functions according to TSH and T4 levels, however that study did not merely include newborn population [1]. Children with DS aged between 5 days and 10 years experienced an increased occurrence of congenital hypothyroidism (with an incidence of 1.8%), and 2 newborns had transient hyperthyrotropinemia [1]. In our study, 2 patients had hypothyroidism and 32 patients had hyperthyrotropinemia in a total of 80 patients.

In a cross-sectional study, Kurtoglu et al. showed that Kayseri was an area of severe iodine deficiency, iodine concentrations in mothers’ urine and breast milk were lower than those of mothers living in iodine sufficient areas, and neonates of these mothers had significantly higher TSH and thyroglobulin levels in comparison to their maternal levels [3]. In the present study hypothyroidism and hyperthyrotropinemia were found in 2 (2.5%) and 32 (40%) of 80 patients, respectively, and iodine deficiency and iodine overload were detected in 12 (42.9%) and 4 (14.2%) of 28 patients, respectively, in whom urinary iodine levels were measured.

In conclusion, TSH elevation in newborns with DS is highly prevalent in the neonatal period. Neonatal screening by thyroid function tests in DS should be performed to prevent further intellectual deterioration and improve overall development. In the neonatal period, hyperthyrotropinemia should be kept in mind and periodical evaluation should be made.

## Competing interests

The authors declare that they have no competing interests.

## Authors’ contributions

DS and SK designed the study and made substantial contribution in drafting the manuscript. DS, MAA, SK, TG, MAO and MA performed acquisition, verification and interpretation of data, and revised the manuscipt critically. All authors read and approved the final manuscript.
